# A new polymorph of *catena*-poly[[tri­aqua­cadmium(II)]-μ_2_-pyrazine-2,3-dicarboxyl­ato]

**DOI:** 10.1107/S1600536809028268

**Published:** 2009-07-22

**Authors:** Hua Yin

**Affiliations:** a Instrumental Measurement and Analysis Center, Fuzhou University, Fuzhou, Fujian 350002, People’s Republic of China

## Abstract

The title complex, [Cd(C_6_H_2_N_2_O_4_)(H_2_O)_3_]_*n*_, is a new monoclinic polymorph. The ortho­rhom­bic form has previously been reported [Ma *et al.* (2006 ▶). *Acta Cryst.* E**62**, m2528–m2529]. The Cd—N and Cd—O bond lengths range from 2.265 (3) to 2.333 (3) Å; a weak Cd—O inter­action is also present, the inter­atomic distance being 2.658 (4) Å. The Cd^II^ ions, which have a distorted penta­gonal-bipyramidal geometry, are bridged by pyrazine-2,3-dicarboxyl­ato ligands, forming a zigzag chain structure. The chains are connected by O—H⋯O hydrogen bonds into a three-dimensional framework.

## Related literature

For the ortho­rhom­bic polymorph, see: Ma *et al.* (2006 ▶). For general background and related structures, see: Mao *et al.* (1996 ▶); Kitaura *et al.* (2002 ▶); Maji *et al.* (2004 ▶); Yin & Liu (2007 ▶, 2009 ▶).
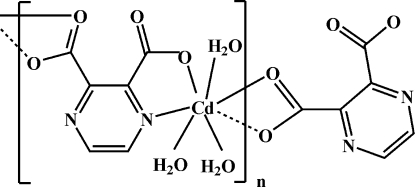


## Experimental

### 

#### Crystal data

[Cd(C_6_H_2_N_2_O_4_)(H_2_O)_3_]
*M* *_r_* = 332.54
Monoclinic, 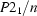

*a* = 5.586 (6) Å
*b* = 15.748 (9) Å
*c* = 10.832 (6) Å
β = 93.94 (3)°
*V* = 950.5 (12) Å^3^
*Z* = 4
Mo *K*α radiation
μ = 2.32 mm^−1^
*T* = 293 K
0.25 × 0.23 × 0.18 mm

#### Data collection

Rigaku Weissenberg IP diffractometer
Absorption correction: multi-scan (*TEXRAY*; Molecular Structure Corporation, 1999 ▶) *T*_min_ = 0.668, *T*_max_ = 1.000 (expected range = 0.440–0.658)
5659 measured reflections
2129 independent reflections
1699 reflections with *I* > 2σ(*I*)
*R*_int_ = 0.032

#### Refinement

*R*[*F* ^2^ > 2σ(*F* ^2^)] = 0.030
*wR*(*F* ^2^) = 0.074
*S* = 1.03
2129 reflections
145 parameters
9 restraints
H-atom parameters constrained
Δρ_max_ = 0.82 e Å^−3^
Δρ_min_ = −0.53 e Å^−3^

### 

Data collection: *TEXRAY* (Molecular Structure Corporation, 1999 ▶); cell refinement: *TEXRAY*; data reduction: *TEXSAN* (Molecular Structure Corporation, 1999 ▶); program(s) used to solve structure: *SHELXS97* (Sheldrick, 2008 ▶); program(s) used to refine structure: *SHELXL97* (Sheldrick, 2008 ▶); molecular graphics: *ORTEX* (McArdle, 1995 ▶); software used to prepare material for publication: *SHELXL97*.

## Supplementary Material

Crystal structure: contains datablocks I, global. DOI: 10.1107/S1600536809028268/bt5003sup1.cif

Structure factors: contains datablocks I. DOI: 10.1107/S1600536809028268/bt5003Isup2.hkl

Additional supplementary materials:  crystallographic information; 3D view; checkCIF report

## Figures and Tables

**Table 1:** Selected geometric parameters (Å, °)

Cd1—O3*W*	2.218 (3)
Cd1—O1	2.265 (3)
Cd1—O3^i^	2.294 (3)
Cd1—N1	2.334 (3)
Cd1—O2*W*	2.397 (4)
Cd1—O1*W*	2.451 (3)

Symmetry code: (i) 
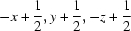
.

**Table 2:** Hydrogen-bond geometry (Å, °)

*D*—H⋯*A*	*D*—H	H⋯*A*	*D*⋯*A*	*D*—H⋯*A*
O1*W*—H1*WA*⋯O4^ii^	0.82	2.56	3.328 (5)	156
O2*W*—H2*WA*⋯O1^iii^	0.84	2.02	2.834 (5)	164
O2*W*—H2*WB*⋯N2^iv^	0.83	2.24	3.036 (4)	161
O3*W*—H3*WA*⋯O4^iv^	0.85	1.82	2.656 (6)	169
O3*W*—H3*WB*⋯O4^v^	0.83	2.25	2.865 (4)	132
O3*W*—H3*WB*⋯O2^v^	0.83	2.22	2.935 (4)	144

Symmetry codes: (ii) 
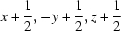
; (iii) 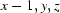; (iv) 
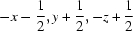
; (v) 
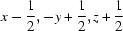
.

**Fig. 1.**
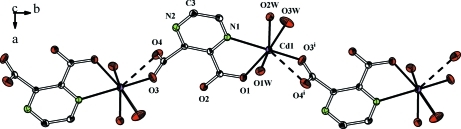
View of the title compound, showing 30% probability displacement ellipsoids. H atoms have been omitted for clarity. [Symmetry code: (i) -x + 1/2, y + 1/2, -z + 1/2.]

## supplementary crystallographic information

### Comment

Pyrazine-2,3-dicarboxylic acid (pzdcH_2_) as a multidentate bridging ligand has been widely applied to construct polymeric coordination compounds (Mao  *et al.*,1996; Kitaura  *et al.*, 2002). Recently, some pzdc-Cd complexes that exhibit selective gas adsorption and photoluminescence were synthesized by self assembling (Maji *et al.*, 2004; Yin *et al.*, 2007; Yin *et al.*, 2009). In this work, a new polymorph of the title compound was obtained by introducing 1,2,4-triazol as the acidity modulating reagent in the reaction of pzdcH_2_ and Cd(II) ion.

The title compound is a one-dimensional zigzag chain structure composed of Cd^II^ ions bridged by pzdc ligands. Cd1 is coordinated by N1, O1 and O3^i^ atoms from two pzdc ligands [symmetry code (i), -*x* + 1/2, *y* + 1/2, -*z* + 1/2] and three coordinated water molecules (O1W, O2W and O3W) (see Fig. 1). The corresponding bond lengths range from 2.265 (3) to 2.333 (3) Å. The Cd1 atom has a weak interaction with O4^i^ (Cd1···O4^i^ = 2.658 (4) Å). Overall, the geometry of Cd may be considered as a distorted pentagonal bipyramid. The title compound crystallizes in the  monoclinic space group P2_1_/n whereas the already known polymorph crystallizes in the orthorhombic   space group P2_1_2_1_2_1_ (Ma *et al.*, 2006). O-H···O hydrogen bonds combine the chains into a three-dimensional framework (Table 2). The neighboring Cd···Cd distance is 8.25 (1) Å.

### Experimental

By adding an aqueous solution of Cd(OAc)_2_ (0.1 mmol) into an aqueous solution of pzdcH_2_(0.1 mmol) and 1,2,4-triazol (0.1 mmol), the resulting mixture was stirred for 2 min and filtered immediately. The colorless crystals of the title compound I were isolated after slow evaporation for 3 days. Yield: *ca*43% based on Cd. Anal. Calcd for C_6_H_8_N_2_O_7_Cd: C, 21.65, H, 2.41, N, 8.42; found: C, 21.58, H, 2.36, N, 8.53.

### Refinement

H atoms of water molecules were located in difference Fourier maps and were then constrained to ride on their parent atoms, with *U*_iso_(H) = 1.5U_eq_(O). Other H atoms were positioned geometrically and constrained to ride on their parent atoms, with C—H = 0.93Å and *U*_iso_(H) = 1.2U_eq_(C). The water H atoms were refined with  distance restraints of O-H 0.86 (2)Å and H-H = 1.38 (2)Å.

### Crystal data

**Table:**

[Cd(C_6_H_2_N_2_O_4_)(H_2_O)_3_]	*F*(000) = 648
*M**_r_* = 332.54	*D*_x_ = 2.324 Mg m^−^^3^
Monoclinic, *P*2_1_/*n*	Mo *K*α radiation, λ = 0.71073 Å
Hall symbol: -P 2yn	Cell parameters from 4538 reflections
*a* = 5.586 (6) Å	θ = 3.2–27.5°
*b* = 15.748 (9) Å	µ = 2.32 mm^−^^1^
*c* = 10.832 (6) Å	*T* = 293 K
β = 93.94 (3)°	Block, colourless
*V* = 950.5 (12) Å^3^	0.25 × 0.23 × 0.18 mm
*Z* = 4	

### Data collection

**Table:**

Weissenberg IP diffractometer	2129 independent reflections
Radiation source: rotor target	1699 reflections with *I* > 2σ(*I*)
graphite	*R*_int_ = 0.032
ω scans	θ_max_ = 27.5°, θ_min_ = 3.2°
Absorption correction: multi-scan (*TEXRAY*; Molecular Structure Corporation, 1999)	*h* = −7→5
*T*_min_ = 0.668, *T*_max_ = 1.000	*k* = −19→20
5659 measured reflections	*l* = −14→14

### Refinement

**Table:**

Refinement on *F*^2^	Primary atom site location: structure-invariant direct methods
Least-squares matrix: full	Secondary atom site location: difference Fourier map
*R*[*F*^2^ > 2σ(*F*^2^)] = 0.030	Hydrogen site location: inferred from neighbouring sites
*wR*(*F*^2^) = 0.074	H-atom parameters constrained
*S* = 1.03	*w* = 1/[σ^2^(*F*_o_^2^) + (0.0366*P*)^2^ + 0.562*P*] where *P* = (*F*_o_^2^ + 2*F*_c_^2^)/3
2129 reflections	(Δ/σ)_max_ < 0.001
145 parameters	Δρ_max_ = 0.82 e Å^−^^3^
9 restraints	Δρ_min_ = −0.53 e Å^−^^3^

### Special details

**Table:**

Geometry. All e.s.d.'s (except the e.s.d. in the dihedral angle between two l.s. planes) are estimated using the full covariance matrix. The cell e.s.d.'s are taken into account individually in the estimation of e.s.d.'s in distances, angles and torsion angles; correlations between e.s.d.'s in cell parameters are only used when they are defined by crystal symmetry. An approximate (isotropic) treatment of cell e.s.d.'s is used for estimating e.s.d.'s involving l.s. planes.
Refinement. Refinement of *F*^2^ against ALL reflections. The weighted *R*-factor *wR* and goodness of fit *S* are based on *F*^2^, conventional *R*-factors *R* are based on *F*, with *F* set to zero for negative *F*^2^. The threshold expression of *F*^2^ > σ(*F*^2^) is used only for calculating *R*-factors(gt) *etc*. and is not relevant to the choice of reflections for refinement. *R*-factors based on *F*^2^ are statistically about twice as large as those based on *F*, and *R*- factors based on ALL data will be even larger.

### Fractional atomic coordinates and isotropic or equivalent isotropic displacement parameters (Å^2^)

**Table:**

	*x*	*y*	*z*	*U*_iso_*/*U*_eq_	
Cd1	0.07189 (6)	0.372955 (14)	0.31158 (2)	0.02540 (10)	
O1	0.3289 (6)	0.28668 (14)	0.2181 (2)	0.0294 (6)	
O2	0.4160 (6)	0.15343 (17)	0.1696 (2)	0.0319 (7)	
O3	0.3399 (7)	−0.01740 (17)	0.3172 (3)	0.0434 (8)	
O4	0.1098 (7)	−0.00334 (19)	0.1449 (3)	0.0475 (9)	
O1W	0.2872 (7)	0.34613 (18)	0.5126 (2)	0.0374 (7)	
H1WA	0.3989	0.3785	0.5318	0.056*	
H1WB	0.1887	0.3401	0.5663	0.056*	
O2W	−0.2530 (6)	0.37407 (15)	0.1551 (3)	0.0358 (7)	
H2WA	−0.3683	0.3410	0.1640	0.054*	
H2WB	−0.3071	0.4212	0.1323	0.054*	
O3W	−0.1846 (8)	0.4335 (2)	0.4320 (3)	0.0583 (11)	
H3WA	−0.3095	0.4581	0.4013	0.088*	
H3WB	−0.1945	0.4277	0.5076	0.088*	
N1	−0.0445 (7)	0.23327 (16)	0.3472 (3)	0.0218 (7)	
N2	−0.1324 (7)	0.06150 (18)	0.3781 (3)	0.0253 (7)	
C1	0.0905 (7)	0.17535 (19)	0.2954 (3)	0.0194 (7)	
C2	0.0436 (7)	0.0893 (2)	0.3109 (3)	0.0195 (7)	
C3	−0.2626 (8)	0.1201 (2)	0.4287 (3)	0.0269 (8)	
H3A	−0.3866	0.1031	0.4763	0.032*	
C4	−0.2215 (8)	0.2060 (2)	0.4137 (3)	0.0242 (8)	
H4A	−0.3187	0.2453	0.4505	0.029*	
C5	0.2939 (8)	0.2074 (2)	0.2211 (3)	0.0216 (7)	
C6	0.1817 (9)	0.0189 (2)	0.2531 (4)	0.0279 (9)	

### Atomic displacement parameters (Å^2^)

**Table:**

	*U*^11^	*U*^22^	*U*^33^	*U*^12^	*U*^13^	*U*^23^
Cd1	0.02614 (19)	0.01735 (14)	0.03381 (15)	0.00158 (10)	0.00999 (10)	0.00080 (10)
O1	0.029 (2)	0.0203 (10)	0.0414 (13)	−0.0017 (11)	0.0180 (12)	−0.0012 (10)
O2	0.027 (2)	0.0258 (12)	0.0445 (15)	−0.0003 (11)	0.0184 (13)	−0.0045 (11)
O3	0.043 (3)	0.0278 (13)	0.0605 (19)	0.0147 (14)	0.0148 (16)	0.0078 (13)
O4	0.044 (3)	0.0526 (17)	0.0482 (16)	−0.0186 (16)	0.0185 (15)	−0.0300 (14)
O1W	0.034 (2)	0.0431 (14)	0.0351 (14)	−0.0041 (13)	0.0042 (12)	0.0050 (12)
O2W	0.031 (2)	0.0274 (12)	0.0480 (15)	−0.0044 (12)	−0.0024 (13)	0.0038 (11)
O3W	0.053 (3)	0.089 (3)	0.0332 (14)	0.035 (2)	0.0059 (15)	−0.0104 (16)
N1	0.023 (2)	0.0185 (12)	0.0247 (13)	−0.0002 (11)	0.0056 (12)	−0.0004 (11)
N2	0.026 (2)	0.0217 (13)	0.0280 (14)	−0.0012 (12)	0.0035 (13)	0.0010 (11)
C1	0.022 (2)	0.0166 (14)	0.0198 (14)	0.0019 (13)	−0.0002 (13)	−0.0009 (12)
C2	0.017 (2)	0.0188 (14)	0.0233 (14)	0.0010 (13)	0.0038 (13)	0.0013 (12)
C3	0.024 (2)	0.0299 (17)	0.0273 (16)	−0.0027 (15)	0.0085 (14)	−0.0003 (14)
C4	0.022 (2)	0.0262 (16)	0.0253 (15)	0.0031 (14)	0.0095 (14)	−0.0030 (13)
C5	0.021 (2)	0.0202 (14)	0.0236 (14)	0.0020 (14)	0.0031 (14)	−0.0020 (13)
C6	0.025 (3)	0.0163 (14)	0.044 (2)	−0.0037 (15)	0.0156 (17)	−0.0055 (15)

### Geometric parameters (Å, °)

**Table:**

Cd1—O3W	2.218 (3)	O2W—H2WB	0.8324
Cd1—O1	2.265 (3)	O3W—H3WA	0.8463
Cd1—O3^i^	2.294 (3)	O3W—H3WB	0.8295
Cd1—N1	2.334 (3)	N1—C1	1.332 (4)
Cd1—O2W	2.397 (4)	N1—C4	1.334 (5)
Cd1—O1W	2.451 (3)	N2—C3	1.317 (5)
O1—C5	1.265 (4)	N2—C2	1.337 (5)
O2—C5	1.245 (4)	C1—C2	1.393 (5)
O3—C6	1.226 (6)	C1—C5	1.522 (5)
O3—Cd1^ii^	2.294 (3)	C2—C6	1.510 (5)
O4—C6	1.262 (5)	C3—C4	1.384 (5)
O1W—H1WA	0.8214	C3—H3A	0.9300
O1W—H1WB	0.8327	C4—H4A	0.9300
O2W—H2WA	0.8385		
			
O3W—Cd1—O1	167.27 (11)	Cd1—O3W—H3WB	128.5
O3W—Cd1—O3^i^	102.02 (13)	H3WA—O3W—H3WB	109.3
O1—Cd1—O3^i^	90.61 (11)	C1—N1—C4	118.0 (3)
O3W—Cd1—N1	96.29 (14)	C1—N1—Cd1	113.8 (2)
O1—Cd1—N1	72.59 (11)	C4—N1—Cd1	128.2 (2)
O3^i^—Cd1—N1	152.11 (10)	C3—N2—C2	116.5 (3)
O3W—Cd1—O2W	85.67 (14)	N1—C1—C2	119.9 (3)
O1—Cd1—O2W	99.25 (12)	N1—C1—C5	117.4 (3)
O3^i^—Cd1—O2W	75.44 (12)	C2—C1—C5	122.6 (3)
N1—Cd1—O2W	85.28 (11)	N2—C2—C1	122.4 (3)
O3W—Cd1—O1W	81.30 (13)	N2—C2—C6	113.7 (3)
O1—Cd1—O1W	90.37 (12)	C1—C2—C6	124.0 (3)
O3^i^—Cd1—O1W	123.85 (12)	N2—C3—C4	122.4 (4)
N1—Cd1—O1W	79.40 (11)	N2—C3—H3A	118.8
O2W—Cd1—O1W	158.60 (11)	C4—C3—H3A	118.8
C5—O1—Cd1	118.5 (2)	N1—C4—C3	120.9 (3)
C6—O3—Cd1^ii^	100.6 (2)	N1—C4—H4A	119.6
Cd1—O1W—H1WA	115.8	C3—C4—H4A	119.6
Cd1—O1W—H1WB	109.4	O2—C5—O1	124.9 (3)
H1WA—O1W—H1WB	114.9	O2—C5—C1	117.5 (3)
Cd1—O2W—H2WA	117.7	O1—C5—C1	117.6 (3)
Cd1—O2W—H2WB	117.3	O3—C6—O4	124.5 (4)
H2WA—O2W—H2WB	108.8	O3—C6—C2	118.4 (3)
Cd1—O3W—H3WA	120.9	O4—C6—C2	116.7 (4)
			
O3W—Cd1—O1—C5	32.7 (8)	N1—C1—C2—N2	−0.9 (6)
O3^i^—Cd1—O1—C5	−154.6 (3)	C5—C1—C2—N2	178.7 (3)
N1—Cd1—O1—C5	2.8 (3)	N1—C1—C2—C6	178.3 (3)
O2W—Cd1—O1—C5	−79.2 (3)	C5—C1—C2—C6	−2.1 (6)
O1W—Cd1—O1—C5	81.6 (3)	C2—N2—C3—C4	0.1 (6)
O3W—Cd1—N1—C1	−174.6 (3)	C1—N1—C4—C3	0.3 (6)
O1—Cd1—N1—C1	−0.9 (2)	Cd1—N1—C4—C3	−178.0 (3)
O3^i^—Cd1—N1—C1	54.4 (4)	N2—C3—C4—N1	−0.5 (6)
O2W—Cd1—N1—C1	100.3 (3)	Cd1—O1—C5—O2	177.2 (3)
O1W—Cd1—N1—C1	−94.7 (3)	Cd1—O1—C5—C1	−4.1 (4)
O3W—Cd1—N1—C4	3.8 (4)	N1—C1—C5—O2	−178.0 (3)
O1—Cd1—N1—C4	177.4 (4)	C2—C1—C5—O2	2.4 (5)
O3^i^—Cd1—N1—C4	−127.3 (3)	N1—C1—C5—O1	3.3 (5)
O2W—Cd1—N1—C4	−81.3 (3)	C2—C1—C5—O1	−176.4 (4)
O1W—Cd1—N1—C4	83.7 (3)	Cd1^ii^—O3—C6—O4	−2.1 (5)
C4—N1—C1—C2	0.4 (5)	Cd1^ii^—O3—C6—C2	171.1 (3)
Cd1—N1—C1—C2	178.9 (3)	N2—C2—C6—O3	−81.2 (5)
C4—N1—C1—C5	−179.2 (3)	C1—C2—C6—O3	99.6 (5)
Cd1—N1—C1—C5	−0.7 (4)	N2—C2—C6—O4	92.5 (4)
C3—N2—C2—C1	0.6 (6)	C1—C2—C6—O4	−86.7 (5)
C3—N2—C2—C6	−178.6 (3)		

Symmetry codes: (i) −*x*+1/2, *y*+1/2, −*z*+1/2; (ii) −*x*+1/2, *y*−1/2, −*z*+1/2.

### Hydrogen-bond geometry (Å, °)

**Table:**

*D*—H···*A*	*D*—H	H···*A*	*D*···*A*	*D*—H···*A*
O1W—H1WA···O4^iii^	0.82	2.56	3.328 (5)	156
O2W—H2WA···O1^iv^	0.84	2.02	2.834 (5)	164
O2W—H2WB···N2^v^	0.83	2.24	3.036 (4)	161
O3W—H3WA···O4^v^	0.85	1.82	2.656 (6)	169
O3W—H3WB···O4^vi^	0.83	2.25	2.865 (4)	132
O3W—H3WB···O2^vi^	0.83	2.22	2.935 (4)	144

Symmetry codes: (iii) *x*+1/2, −*y*+1/2, *z*+1/2; (iv) *x*−1, *y*, *z*; (v) −*x*−1/2, *y*+1/2, −*z*+1/2; (vi) *x*−1/2, −*y*+1/2, *z*+1/2.
